# Detecting Cognitive Decline and Dementia in Santa Cruz, Galápagos Islands, Ecuador

**DOI:** 10.7759/cureus.10826

**Published:** 2020-10-06

**Authors:** Patricio H Espinosa del Pozo, Patricio S Espinosa, Eduardo Donadi, Lenin Rogel, Raquel Naranjo, Gabriela E Haro, Diana C Riera, Gabriela S Mendoza, Nicole A Garzón, Nicolas F Andrade

**Affiliations:** 1 Neuroscience, Universidad Central del Ecuador, Quito, ECU; 2 Neurology, Marcus Neuroscience Institute, Boca Raton Regional Hospital Baptist Health System, Boca Raton, USA; 3 Departamento De Clinica, Facultad De Medicina Ribeirão Preto, Universidad De São Paulo, Ribeirão Preto, BRA; 4 Educación y Ciencias Sociales, Universidad Central del Ecuador, Santa Cruz, ECU; 5 Neurosciences Unit, College of Medical Sciences, Central University of Ecuador, Quito, ECU; 6 Biological Sciences, Webster University, St. Louis, USA

**Keywords:** galapagos, cognitive decline, alzheimer’s dementia, dementia, memory loss

## Abstract

Objective

To assess the cognitive function, prevalence, and risk factors associated with cognitive decline and dementia in people above 65 years of age in Santa Cruz Island, Galápagos, Ecuador.

Methods

This is a cross-sectional observational study that was carried out in adults over 65 years of age in Santa Cruz Island, Galápagos, Ecuador. The mini-mental state examination (MMSE) and ascertain dementia eight-item informant questionnaire (AD8)-validated Ecuador Spanish versions were used to assess cognition.

Results

There were a total of 80 participants, 55 (67%) women and 25 (31.2%) men. The majority of participants were Mestizos (85.3%), with the remainder classified as White (4.8%), Afro-Ecuadorians (2.4%), or Indigenous (3.6%). The prevalence of cognitive impairment is 30.0%-43.7%. The MMSE results showed that older age and lack of education are risk factors for cognitive decline (p < 0.01). There was high correlation between MMSE and AD8 scores. The AD8 showed that older age, widowhood, and living in Santa Rosa were risk factors for cognitive decline (p < 0.01). According to the AD8, the group with the highest education (six years or more) had the lowest risk of cognitive decline and dementia (p < 0.01).

Conclusions

The main risk factors for cognitive decline and dementia in individuals above 65 years old in Santa Cruz Island, Galápagos, Ecuador are increased age, lack of education, and widowhood. The prevalence of cognitive impairment is similar to previous studies in Ecuador.

## Introduction

In Ecuador, our group in 2018 in the province of Pichincha reported a prevalence of cognitive impairment and dementia of 18%-22% at age 65 and 54%-60% at age 80, which is higher than developed countries and consistent with data of other developing countries [1-3]. To better understand the differences between regions of Ecuador, we conducted a study to determine the risk factors and prevalence of cognitive decline and dementia in the Island of Santa Cruz, Province of Galápagos, Ecuador. We chose this island due to its highest population concentration and mainly because of the local community and political leaders' generous support.

The Galápagos Islands are an archipelago of volcanic islands located in the Pacific Ocean approximately 1,000 km (621 mi). It is traversed by the equatorial line and west of continental Ecuador in South America. The Galápagos Islands are a province of the Republic of Ecuador, world-renowned for their large number of endemic species and where Charles Darwin research resulted in Darwin's theory of evolution of the species. The Galápagos Islands is a protected national park that is composed of over 100 islands of various sizes and its surrounding waters. Migration to the island is very limited to preserve the ecosystem. The population in this island has increased at a very slow pace for the last 70 years. In 1950, there were 1,346 inhabitants, and the population increased at a rate of 4%-6% per decade until the population raised to 17,451 inhabitants and Ecuador in the year 2001 imposed a policy that limited the migration. Due to this measure, the population growth has decreased to 1.8% per decade in the last decade. According to the national census of 2015, the population of the Galápagos Islands is 25,244 inhabitants. The population is divided among three islands: Santa Cruz with 15,701 habitants, San Cristóbal with 7,199 habitants, and Isabela with 2,344 inhabitants. According to the census data, the mean age of the population is 29.3 years, 36% of the population were born in the island, and the rest of the population are immigrants from other provinces from Ecuador. The Galápagos Islands' ethnicity is composed of 85% Mestizo, 8% Indígena, 3% Afro-ecuadorian, 3% White, and 1% Montubio.

Our study aimed to determine the prevalence and risk factors of cognitive decline and dementia in this unique, isolated, migration-limited population.

## Materials and methods

This is an observational, descriptive, cross-sectional, and prospective study carried out between January 2019 and January 2020. The study subjects were adults older than 65 years of age living in Santa Cruz Island, Galápagos.

The sample size was calculated according to the Ecuador National Census data of 2015, which reported that the Galápagos Islands have a population of 25,244 individuals, of which 15,701 live in Santa Cruz Island, 918 (5.8%) are older than 60 years of age.

To determine the total sample size, the following parameters were taken into account: 95% confidence interval, 5% margin of error, estimated prevalence of dementia of 0.6, and estimated population size of patients over age 65 of 916. It was determined that a minimum sample of 80 subjects were needed. In this study, we were able to recruit 87 patients, which was above the required minimum. All the patients in this study gave informed consent to participate. The informed consent was approved by the Institutional Review Board of the Escuela de Medicina de la Universidad Central del Ecuador.

The inclusion criteria were age greater than 65 years and signed informed consent to participate. The exclusion criteria were being under 65 years of age and decline to participate in the study. The study participants were selected randomly. The participants were selected from home addresses and care centers for the elderly, which were randomly selected from different neighborhoods of Santa Cruz Island (Bellavista, Puerto Ayora, and Santa Rosa). 

The data was collected in the form of written questionnaires for each of the assessments: mini-mental state examination (MMSE) and ascertain dementia eight-item informant questionnaire (AD8)-validated Ecuador Spanish versions. Description of the scores and interpretation have been reported previously [4]. The study's point prevalence was determined by calculating the proportion of individuals with cognitive decline, identified by the MMSE/AD8, divided by the study sample size. For the statistical analysis, we used the Fisher's exact test to compare differences in gender distribution and cognitive status classes. A logistic regression model was used to analyze the association between AD8 scores and the neighborhoods location (Bella Vista, Puerto Ayora, and Santa Rosa), gender (male/female), age group (years), marital status (married/divorced), ethnical group (Mestizo, White, Afro-Ecuadorian, and Indigenous), and education level (in years of education). A logistic model including all study variables was used to estimate the adjusted odds ratio (OR), considering possible confounder effects [5].

## Results

**Figure 1:**
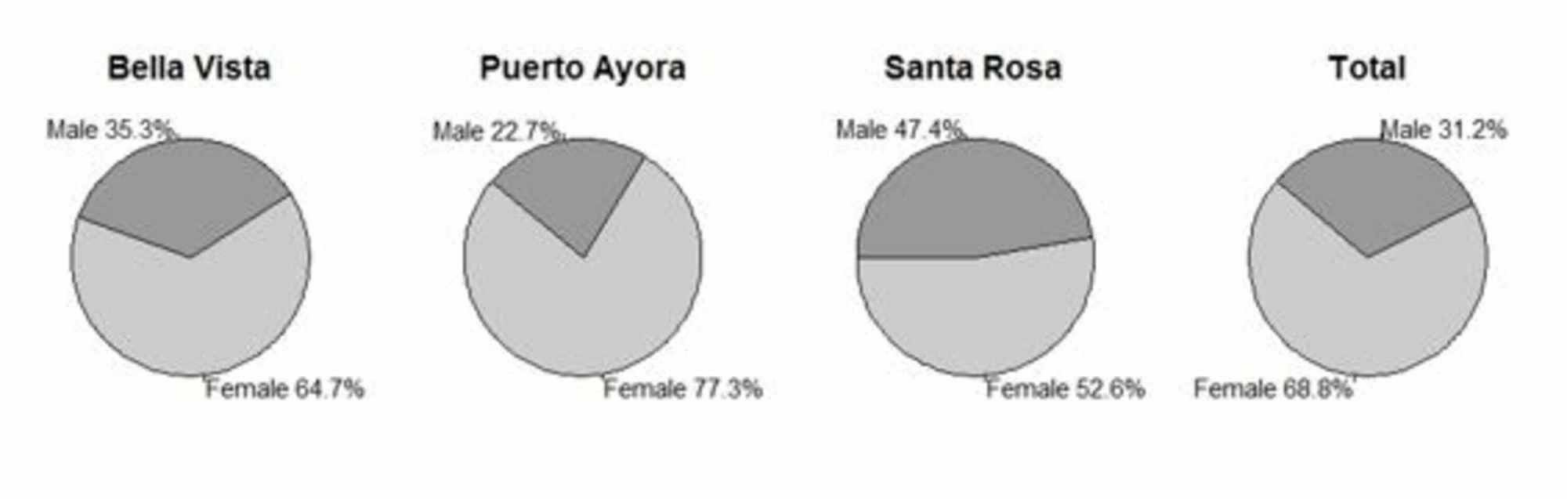
Study gender distribution by neighborhood in Santa Cruz Island, Galápagos.

**Figure 2:**
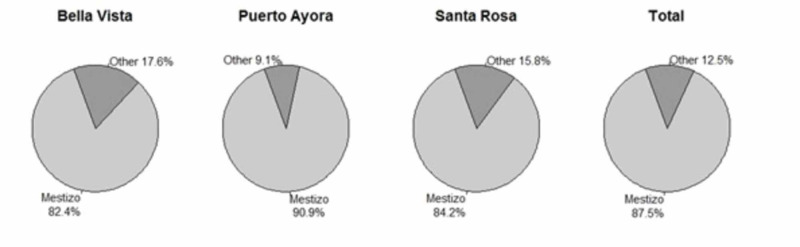
Ethnicity composition in Santa Cruz, Island.

There were 80 participants that fulfilled the inclusion criteria, with 55 (67%) women and 25 (31.2%) men. The majority of participants were Mestizos (85.3%), with the remainder classified as White (4.8%), Afro-Ecuadorian (2.4%), or Indigenous (3.6%) ​(Table 1 and Figures 1, 2).

**Table 1:** Participant demographics, ethnicity, and education by location (n = 80).

		Location			
		Bella Vista (n = 17)	Puerto Ayora (n = 44)	Santa Rosa (n = 19)	Total (n = 80)
Gender	Female	11 (64.7)	34 (77.3)	10 (52.6)	55 (68.8)
	Male	6 (35.3)	10 (22.7)	9 (47.4)	25 (31.2)
Age (years)	65-69	0	8 (18.2)	4 (21.1)	12 (15)
	70-74	3 (17.6)	12 (27.3)	5 (26.3)	20 (25)
	75-79	5 (29.4)	9 (20.5)	6 (31.6)	20 (25)
	80-84	4 (23.5)	7 (15.9)	4 (21.1)	15 (18.8)
	≥85	5 (29.4)	8 (18.2)	0	13 (16.2)
Marital	Married	12 (70.6)	22 (50.0)	14 (73.7)	48 (60)
	Divorced	0	3 (6.8)	0	3 (3.8)
	Single	1 (5.9)	9 (20.5)	3 (15.8)	13 (16.2)
	Widowed	4 (23.5)	10 (22.7)	2 (10.5)	16 (20)
Ethnics	Mestizo	14 (82.4)	40 (90.9)	16 (84.2)	70 (87.5)
	White	1 (5.9)	2 (4.5)	1 (5.3)	4 (5)
	Indian	1 (5.9)	1 (2.3)	1 (5.3)	3 (3.8)
	Afro-Ecuadorian	1 (5.9)	0 (0)	1 (5.3)	2 (2.5)
	Other	0	1 (2.3)	0	1 (1.2)
Education	No education	1 (5.9)	10 (22.7)	0	11 (13.8)
	One to two years	4 (23.5)	3 (6.8)	2 (10.5)	9 (11.2)
	Three to six years	9 (52.9)	13 (29.5)	9 (47.4)	31 (38.8)
	>Six years	3 (17.6)	18 (40.9)	8 (42.1)	29 (36.2)

**Figure 3:**
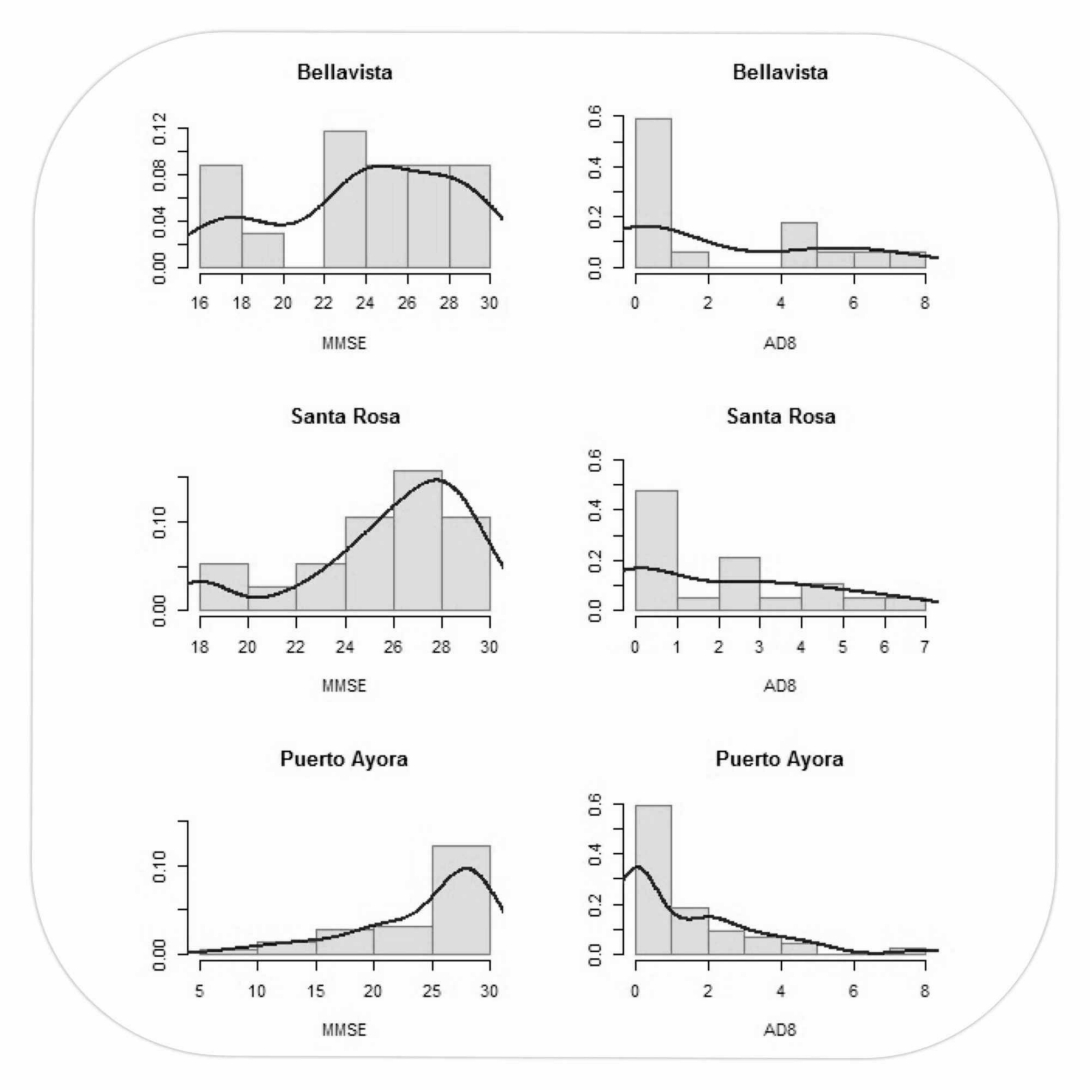
Histogram of MMSE and AD8 scores by neighborhoods of Santa Cruz Island, Galápagos. MMSE: Mini-mental state examination; AD8: ascertain dementia eight-item informant questionnaire.

**Figure 4:**
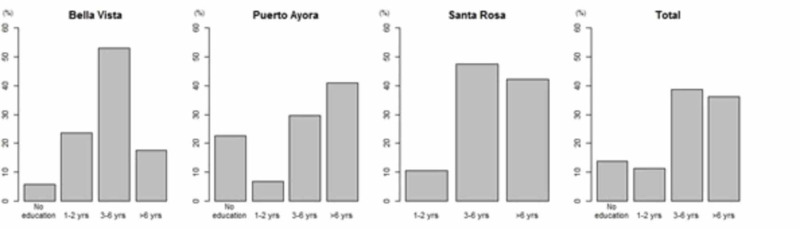
Education of study participants.

This distribution is consistent with the data found in the last Ecuadorian census, in which the majority of people (71.99%) were self-described as Mestizos. In terms of education, 36.2% of participants had more than six years of education, 38.7% of participants had three to six years of education, 11.2% of participants had one to two years of education, and 13.7% had no education. Puerto Ayora had the highest number of individuals with more than six years of education (40.9%). Women as group had fewer years of education than men (see Tables 2, 3 and Figures 2, 3). The MMSE and AD8 scores by neighborhoods of Santa Cruz Island, Galápagos had a similar distribution (see Figure 4).

**Table 2:** Study variables and MMSE scores (n = 80). MMSE: Mini-mental state examination.

		MMSE score			
		Normal (n = 56)	Mild (n = 13)	Moderate or severe (n = 11)	Fisher's exact test (p values)
Location	Bella Vista	10 (17.9)	4 (30.8)	3 (27.3)	0.50
	Puerto Ayora	30 (53.6)	8 (61.5)	6 (54.5)	
	Santa Rosa	16 (28.6)	1 (7.7)	2 (18.2)	
Gender	Female	36 (64.3)	12 (92.3)	7 (63.6)	0.11
	Male	20 (35.7)	1 (7.7)	4 (36.4)	
Age (years)	65-69	12 (21.4)	0	0	0.02
	70-74	14 (25.0)	4 (30.8)	2 (18.2)	
	75-79	14 (25.0)	4 (30.8)	2 (18.2)	
	80-84	12 (21.4)	2 (15.4)	1 (9.1)	
	≥85	4 (7.1)	3 (23.1)	6 (54.5)	
Marital	Married	37 (66.1)	8 (61.5)	3 (27.3)	0.10
	Divorced/Single	11 (19.6)	2 (15.4)	3 (27.3)	
	Widowed	8 (14.3)	3 (23.1)	5 (45.5)	
Ethnics	Mestizo	48 (85.7)	11 (84.6)	11 (100)	0.57
	Other	8 (14.3)	2 (15.4)	0	
Education	No education	1 (1.8)	4 (30.8)	6 (54.5)	<0.01
	One to two years	4 (7.1)	4 (30.8)	1 (9.1)	
	Three to six years	22 (39.3)	5 (38.5)	4 (36.4)	
	>Six years	29 (51.8)	0	0	
AD8	0-1	38 (67.9)	5 (38.5)	2 (18.2)	<0.01
	≥2	18 (32.1)	8 (61.5)	9 (81.8)	

**Table 3:** Participant demographics, ethnicity, and education by gender (n = 80).

		Gender		
		Female (n = 55)	Male (n = 25)	Fisher's exact test (p values)
Age (years)	65-69	10 (18.2)	2 (8.0)	0.21
	70-74	16 (29.1)	4 (16.0)	
	75-79	10 (18.2)	10 (40.0)	
	80-84	11 (20.0)	4 (16.0)	
	>85	8 (14.5)	5 (20.0)	
Marital	Married	31 (56.4)	17 (68.0)	0.29
	Divorced	2 (3.6)	1 (4.0)	
	Single	8 (14.5)	5 (20.0)	
	Widowed	14 (25.5)	2 (8.0)	
Ethnics	Mestizo	47 (85.5)	23 (92.0)	0.49
	Other	8 (14.5)	2 (8.0)	
Education	No education	10 (18.2)	1 (4.0)	0.33
	One to two years	6 (10.9)	3 (12.0)	
	Three to six years	19 (34.5)	12 (48.0)	
	>Six years	20 (36.4)	9 (36.0)	

The MMSE results showed that older age and lack of education are a risk factor for cognitive decline (p < 0.01). At age 85 and above, 54.5% of people in our population showed evidence of cognitive decline. Poor education was also a significant risk factor for cognitive decline and dementia (p < 0.01). The group with no education was observed to contain a higher fraction of individuals with moderate and severe dementia than educated groups. There was a high correlation between MMSE and AD8 scores. We found no statistically significant association between the MMSE and cognitive decline in the following variables: gender, ethnicity, marital status, and location (see Tables 3, 4).

**Table 4:** Comparison between the MMSE and AD8 in the detection of cognitive impairment. MMSE: Mini-mental state examination; AD8: ascertain dementia eight-item informant questionnaire.

Instrument	Total # participants	Normal	Abnormal ( prevalence)
	n	n (%)	n (%)
MMSE	80	56 (70.0)	24 (30.0)
AD8	80	45 (56.2)	35 (43.7)

The results of the AD8 showed that older age, widow status, and living in Santa Rosa were risk factors for cognitive decline (p < 0.01). According to the AD8 the group with the highest education (six years or more) had the lowest risk of cognitive decline and dementia (p < 0.01). We found no statistically significant association between the AD8 and cognitive decline in the following variables: gender, ethnicity, married, and divorced (see Table 5).

**Table 5:** Study variables and AD8 scores. *An asterisk marks significant associations. OR: Odds ratio; CI: confidence interval; AD8: ascertain dementia eight-item informant questionnaire.

		AD8 scores			
		0-1 point (n = 45)	≥2 points (n = 35)	Crude OR (95% CI)	Adj. OR (95% CI)
Location	Bella Vista	10 (22.2)	7 (20.0)	Ref.	Ref.
	Puerto Ayora	26 (57.8)	18 (51.4)	0.9 (0.3-3.1)	1.9 (0.3-9.3)
	Santa Rosa	9 (20.0)	10 (28.6)	1.6 (0.4-5.9)	7.8 (1.2-49.3)*
Gender	Female	28 (62.2)	27 (77.1)	Ref.	Ref.
	Male	17 (37.8)	8 (22.9)	0.5 (0.1-1.3)	0.3 (0.1-1.1)
Age (years)	65-69	10 (22.2)	2 (5.7)	Ref.	Ref.
	70-74	12 (26.7)	8 (22.9)	3.3 (0.5-19.4)	3.5 (0.4-29.4)
	75-79	12 (26.7)	8 (22.9)	3.3 (0.5-19.4)	8.1 (0.7-86.7)
	80-84	7 (15.6)	8 (22.9)	5.7 (0.9-35.5)	15.5 (1.4->100)*
	≥85	4 (8.9)	9 (25.7)	11.2 (1.6-76.8)*	31.7 (1.6->100)*
Marital	Married	28 (62.2)	20 (57.1)	Ref.	Ref.
	Divorced/Single	13 (28.9)	3 (8.6)	0.3 (<0.1-1.2)	0.2 (<0.1-1.0)
	Widowed	4 (8.9)	12 (34.3)	4.2 (1.2-16.8)*	1.5 (0.3-8.0)
Ethnics	Mestizo	37 (82.2)	33 (94.3)	Ref.	Ref.
	Other	8 (17.8)	2 (5.7)	0.3 (<0.1-1.2)	0.2 (<0.1-1.7)
Education	No education	3 (6.7)	8 (22.9)	Ref.	Ref.
	One to two years	6 (13.3)	3 (8.6)	0.2 (<0.1-1.3)	0.2 (<0.1-2.1)
	Three to six years	16 (35.6)	15 (42.9)	0.4 (<0.1-1.6)	0.3 (<0.1-2.3)
	>Six years	20 (44.4)	9 (25.7)	0.2 (<0.1-0.8)*	0.2 (<0.1-1.3)

## Discussion

The estimated prevalence of cognitive impairment in adults over age 65 in Santa Cruz Island, Galápagos, Ecuador, is 30.0% by the MMSE and 43.7% by the AD8. These data are consistent with our previous studies in Ecuador and studies done in other parts of the world [1-3]. The results of this study suggest that both the AD8 and MMSE are valid instruments for identifying cognitive impairment in the Galápagos Islands. Additionally, MMSE scores correlated consistently with AD8 scores. Thus, both exams appear to be reliable tools in assessing cognitive function and screening for cognitive decline. To our knowledge this is the first time that cognitive decline has been formally investigated in this remote, isolated, and unique Ecuadorian population.

Age was observed to be a statistically significant risk factor for cognitive decline and dementia (p < 0.01). The MMSE scores demonstrated cognitive impairment in 18.24% in the 70- to 74-year-old group, and 54.5% in the 85 and over year-old group. The AD8 results showed similar prevalence in each group at 22.9.4% and 25.7%, respectively. These values are somewhat within the range of those reported in the international literature, in which the prevalence of dementia has been reported at 11% at age 65 and 50% at age 85. The AD8 appears to be more sensitive in detecting cognitive decline and dementia than the MMSE, and the results obtained with the AD8 are consistent with those of previous studies [1-4]. 

The results of this study suggest that a low level of education is a major risk factor for cognitive impairment and dementia (p < 0.01) in this population. In Santa Cruz Island, most residents had an education under six years in length (63.7%), 13.7% had no education, 11.2% had one to two years, 38.7% had three to six years, and 36.2% had six or more years of education. Women in our study group tended to have much less education than the men, consistent with prior studies done in Ecuador. This is an important finding since poor education is a well-recognized and modifiable risk factor for dementia worldwide [6].

Ethnicity was not a significant risk factor for cognitive dysfunction and dementia in our sample population. We know that ethnic minority groups such as African Americans, Latinos, and Caribbean Americans have a higher risk of dementia than Caucasians. Possible explanations for the lack of observed difference in the risk between ethnicities in our study maybe due to a small number of individuals of other ethnicities than Mestizos.

The AD8 results show that widowhood (p < 0.01) is a statistically significant risk factor for cognitive impairment and dementia. In other words, patients who become widow are at higher risk for dementia. Previous studies have validated this, which shows that experience of a stressful life event is associated with an increased risk of dementia [7]. Also, the AD8 shows that those individuals with high education (six years or more) were at a lower risk of dementia (p < 0.01). This result is very interesting because it highlights the protective effect of education. 

Our study's limitation is that our instruments are designed to detect cognitive impairment and not to diagnose dementia, which requires more formal physical and neurological examination, imaging studies, and laboratory testing. Another limitation of the study was the sample size. Few participants had moderate to severe cognitive impairment, making it statistically impossible to fit a logistic regression model to analyze the association between MMSE scores and the variables of interest. Although our study may have multiple limitations, our data has external validity because it consistently shows the widely observed correlation between increased age, low education, and cognitive impairment and dementia. 

## Conclusions

The prevalence of cognitive impairment in individuals who are 65 years and older in Santa Cruz, Galápagos Islands, Ecuador, is similar to previous studies in Ecuador and other parts of the world. The main risk factors for cognitive decline and dementia identified in this study population are increased age, lack of education, and widowhood. Based on these findings, opportunities to reduce the incidence of cognitive decline and dementia in Santa Cruz Island, Galápagos, Ecuador, should be sought out, beginning with addressing modifiable risk factors. This may include increasing access to education and encouraging healthy living and well-being. This study shows that the MMSE and AD8 screening tests are effective in detecting cognitive impairment and dementia in the Galápagos Islands.
